# Investigating oral human papillomavirus co-infection with *Neisseria gonorrhoeae* and *Chlamydia trachomatis*

**DOI:** 10.1017/S095026882300198X

**Published:** 2024-01-08

**Authors:** Ella Trembizki, Taylah Anderson, David M. Whiley, Annika Antonsson

**Affiliations:** 1 The University of Queensland Centre for Clinical Research, Faculty of Medicine, The University of Queensland, Brisbane, QLD, Australia; 2 Pathology Queensland Central Laboratory, Brisbane, QLD, Australia; 3 Department of Population Health, QIMR Berghofer Medical Research Institute, Brisbane, QLD, Australia; 4 Faculty of Medicine, The University of Queensland, Brisbane, QLD, Australia

**Keywords:** co-infection, HPV, oral HPV, sexually transmissible infections, epidemiology

## Abstract

Compared to cervical cancer, little is known about human papillomavirus (HPV)-driven oropharyngeal cancer and their cofactors. Here, we investigated potential associations between *Chlamydia trachomatis* (CT) and *Neisseria gonorrhoeae* (NG) with oral HPV and HPV persistence, which are known cofactors in cervical carcinogenesis, and also play a role in HPV-driven oropharyngeal cancer. Saliva samples (*n* = 547) from 312 people were tested for CT and NG and whom had previously been tested for oral HPV infection in a longitudinal study. Eight participants were positive for CT (2.6%) and one for NG (0.3%). Six of these nine participants were also positive for oral HPV in at least one of their samples. We found no significant associations between HPV, CT, or NG infection in the saliva samples analyzed. These preliminary data suggest CT and NG have little influence on oral HPV-positivity and persistence in a general population. However, larger studies focusing on ‘at risk’ population cohorts are necessary to assess potential associations between oral sexually transmissible infections and oral HPV infections, and their outcomes.

## Introduction

There has been a major increase in the number of human papillomavirus (HPV)-positive oropharyngeal squamous cell carcinomas (OSCC) in Australia over the past three decades and this is likely caused by oral HPV infections acquired through oral sex. A subclinical oral HPV infection that persists for decades is likely to precede an HPV-driven OSCC (proven for cervical cancer) [1]. Almost all cervical cancers are HPV-driven, and other sexually transmissible infections (STIs; i.e., *Chlamydia trachomatis* (CT), and *Neisseria gonorrhoeae* (NG)) can act as cofactors in cervical carcinogenesis [2, 3]. Compared to cervical cancer, there are several important research gaps in knowledge of persistent oral HPV infection, HPV-driven oropharyngeal cancer, and their cofactors. STI rates have significantly increased in the past decade and presumably so have oral STIs. Despite this, the prevalence of oral and pharyngeal STIs is currently not well captured, especially in heterosexual populations where pharyngeal testing of NG is not included in the testing guidelines in Australia (except for sex workers). Furthermore, oropharyngeal infections are generally asymptomatic thus contributing to underdiagnosis. Here, as a first step, we sought to explore CT and NG positivity in individuals with and without oral HPV infection using a saliva sample bank collected from the general population [4].

## Methods

Testing was conducted retrospectively on our previously established longitudinal cohort, the Oral Health Study (OHS) [4]. Oral samples and questionnaire data (lifestyle, health, Gardasil vaccination, sexual behaviour, and history) were available for OHS participants. Participants (*n* = 636) donated an oral sample at baseline, 6, 12, and 24 months. Samples were previously tested for HPV (positive samples were HPV typed) as described [4]; samples were extracted using the Promega Maxwell system, HPV was detected with a nested PCR system, and positive samples were typed by direct sequencing.

For this sub-study, we selectively tested two participant groups, (1) at least one HPV-positive oral sample (*n* = 132) and (2) HPV-negative (*n* = 180). In total, 547 samples from 312 people were tested. The participants were recruited from workplaces (*n* = 69), GP clinics (*n* = 127), STI clinics (*n* = 44), and university campuses (*n* = 72). Saliva samples were tested for CT/NG in duplicate on the Cepheid, GeneXpert commercial platform (Cepheid, Sunnyvale, CA). It should be noted that saliva is not a validated sample type for the GeneXpert CT/NG test; hence, this testing was research-use-only.

## Results

Overall, 10 of 547 samples tested for CT/NG did not pass the quality control and were excluded from analyses. Valid results were obtained for the remaining 537 samples (*n* = 312 participants). Nine oral infections were detected; eight positive for CT (2.6%) and one for NG (0.3%). All nine infections were from separate participants, five males and four females, with four recruited from GP clinics, three from universities, and two from sexual health clinics. Further, 6/132 (4.5%) HPV positive participants and 3/180 (1.7%) HPV negative had CT/NG diagnosis (Z score not significant). The six CT/NG participants who were HPV-positive had oral infections with HPV-18, -31, -32, -59, -61, and 62 (HPV-18, -31, and -59 are high-risk HPV types). For association analysis, CT and NG positive samples were pooled into one variable with nine CT/NG positive people. Table 1 summarizes oral HPV positivity and persistence with associations of CT/NG diagnosis. Briefly, six of the nine CT/NG-positive participants were also positive for oral HPV in at least one sample (*n* = 4 with one oral HPV-positive sample and *n* = 2 with two HPV-positive samples over time) while three of the participants with CT/NG positive samples were oral HPV-negative. There were no significant associations found between CT/NG positivity and oral HPV positivity and persistence (Table 1). Supplementary Table 1 shows the associations between CT/NG positivity and sexual behaviour and history variables. Of note, a significant association between CT/NG-positive participants and a previous STI diagnosis was found as well as illicit drug use.

**Table 1.** Oral HPV and CT/NG positivity associations

		CT/NG −	CT/NG +	
	Category	*N* (%)	*N* (%)	*p*-value
Total number of oral HPV-pos samples^a^	0	177 (98.3)	3 (1.7)	
	1	93 (95.9)	4 (4.1)	
	2	25 (92.6)	2 (7.4)	
	3	6 (100)	0 (0.0)	
	4	2 (100)	0 (0.0)	0.449
Oral HPV incidence^a^	No	92 (96.8)	3 (3.2)	
	Yes	84 (96.6)	3 (3.4)	0.913
Oral HPV clearance^a^	No	23 (95.8)	1 (4.2)	
	Yes	59 (95.2)	3 (4.8)	0.894
Oral HPV persistence^a^	No	58 (95.1)	3 (4.9)	
	Yes	24 (96.0)	1 (4.0)	0.854

a Data from the Oral Health Study [4].

## Discussion

It has long been established that HPV plays a central role in cervical carcinogenesis, but also that additional cervical STIs such as CT and NG could increase the risk of developing cervical cancer [2, 3]. Here, we explored the associations of CT or NG infections with oral HPV in a primarily random population cohort within the OHS study and observed very low CT/NG positivity. We observed 4.5% of HPV positive and 1.7% HPV negative participants harboured CT/NG in their saliva. These were non-significant associations between oral HPV infection, persistence, and CT or NG infection, albeit acknowledging that this sample size was too small to ascertain associations comprehensively. Research on the persistence of STIs in the oral cavity and associated impact upon HPV is limited. These pilot data, while suggesting that CT and NG have little influence on HPV positivity and persistence, could help inform future larger studies assessing potential associations between oral STIs and oral HPV infections and their outcomes.

## Supporting information

Trembizki et al. supplementary material Trembizki et al. supplementary material

## Data Availability

The data that support the findings of this study are available from the corresponding author upon reasonable request.

## Abbreviations

CT: *Chlamydia trachomatis*
HPV: human papillomavirus
NG: *Neisseria gonorrhoeae*
OHS: the Oral Health Study
STI: sexually transmissible infection

## Supplementary material

For supplementary material accompanying this paper visit http://doi.org/10.1017/S095026882300198X. click here to view supplementary material
