# Supplementary material for: Investigating oral human papillomavirus co-infection with Neisseria gonorrhoeae and Chlamydia trachomatis
Source: Epidemiol Infect. 2024 Jan 8;152:e8. doi: 10.1017/S095026882300198X (PMC10789976; doi:10.1017/S095026882300198X)
Supplement: Trembizki et al. supplementary material [file S095026882300198Xsup001.docx]

| Supplementary Table 1.  Sexual history and behaviour data for participants. | | **CT/NG -** | **CT/NG +** |  |
| --- | --- | --- | --- | --- |
|  | **Category** | **N (%)** | **N (%)** | **P value**** |
| Lifetime number of Kissing Partners | 0-3 | 70 (97.2) | 2 ( 2.8) |  |
|  | 4-7 | 54 ( 100) | 0 ( 0.0) |  |
|  | 8-15 | 62 (96.9) | 2 ( 3.1) |  |
|  | 16-28 | 52 (98.1) | 1 ( 1.9) |  |
|  | 29+ | 65 (94.2) | 4 ( 5.8) | 0.420 |
| Lifetime sexual intercourse partners | None | 13 (92.9) | 1 ( 7.1) |  |
|  | 1 | 32 ( 100) | 0 ( 0.0) |  |
|  | 2-3 | 45 ( 100) | 0 ( 0.0) |  |
|  | 4-7 | 72 (98.6) | 1 ( 1.4) |  |
|  | 8-15 | 60 (96.8) | 2 ( 3.2) |  |
|  | 16 or more | 64 (94.1) | 4 ( 5.9) | 0.276 |
| Ever given oral sex | No | 27 (96.4) | 1 ( 3.6) |  |
|  | Yes | 265 (97.1) | 8 ( 2.9) | 0.881 |
| Lifetime Given Oral Sex Partners | None | 27 (96.4) | 1 ( 3.6) |  |
|  | 1 | 35 ( 100) | 0 ( 0.0) |  |
|  | 2-3 | 80 (98.8) | 1 ( 1.2) |  |
|  | 4-7 | 66 (97.1) | 2 ( 2.9) |  |
|  | 8-15 | 32 ( 100) | 0 ( 0.0) |  |
|  | 16 or more | 46 (92.0) | 4 ( 8.0) | 0.162 |
| Ever Received Oral Sex | No | 26 (96.3) | 1 ( 3.7) |  |
|  | Yes | 264 (97.1) | 8 ( 2.9) | 0.875 |
| Lifetime Received Oral Sex Partners | None | 26 (96.3) | 1 ( 3.7) |  |
|  | 1 | 30 ( 100) | 0 ( 0.0) |  |
|  | 2-3 | 83 ( 100) | 0 ( 0.0) |  |
|  | 4-7 | 64 (95.5) | 3 ( 4.5) |  |
|  | 8-15 | 38 ( 100) | 0 ( 0.0) |  |
|  | 16 or more | 43 (91.5) | 4 ( 8.5) | 0.052 |
| Preferred Gender(s) | Same sex only | 14 (93.3) | 1 ( 6.7) |  |
|  | Opposite sex only | 239 (98.0) | 5 ( 2.0) |  |
|  | Both sexes | 38 (92.7) | 3 ( 7.3) | 0.130 |
| Ever oral HPV positive* | yes | 126 (95.5) | 6 ( 4.5) |  |
|  | no | 177 (98.3) | 3 ( 1.7) | 0.133 |
| Previous STI (diagnosed by doctor) | no | 226 (98.3) | 4 ( 1.7) |  |
|  | yes | 77 (93.9) | 5 ( 6.1) | **0.043** |
| Previous Chlamydia infection | No | 278 (98.2) | 5 ( 1.8) |  |
|  | Yes | 25 (86.2) | 4 (13.8) | **0.0002** |
| Previous Gonorrhoea infection | No | 289 (97.0) | 9 ( 3.0) |  |
|  | Yes | 14 ( 100) | 0 ( 0.0) | 0.509 |
| Previous Syphilis infection | No | 299 (97.1) | 9 ( 2.9) |  |
|  | Yes | 4 ( 100) | 0 ( 0.0) | 0.729 |
| Previous HPV infection | No | 275 (97.2) | 8 ( 2.8) |  |
|  | Yes | 28 (96.6) | 1 ( 3.4) | 0.849 |
| Previous HSV infection | No | 282 (96.9) | 9 ( 3.1) |  |
|  | Yes | 21 ( 100) | 0 ( 0.0) | 0.413 |
| Previous HIV infection | No | 298 (97.1) | 9 ( 2.9) |  |
|  | Yes | 5 ( 100) | 0 ( 0.0) | 0.698 |
| Protection during Oral Sex | Never | 169 (97.1) | 5 ( 2.9) |  |
|  | Occasionally | 42 ( 100) | 0 ( 0.0) |  |
|  | Frequently | 7 ( 100) | 0 ( 0.0) |  |
|  | Prefer not to answer | 85 (95.5) | 4 ( 4.5) | 0.517 |
| Smoking status*** | Current smoker | 25 (92.6) | 2 ( 7.4) |  |
|  | Ex-smoker | 86 (95.6) | 4 ( 4.4) |  |
|  | Never smoker | 192 (99.0) | 2 ( 1.0) | 0.060 |
| Alcohol consumption (standard drinks per week)**** | None | 29 (96.7) | 1 ( 3.3) |  |
|  | <1 | 90 (95.7) | 4 ( 4.3) |  |
|  | 2-4 | 75 (98.7) | 1 ( 1.3) |  |
|  | 5-13 | 81 (98.8) | 1 ( 1.2) |  |
|  | 14+ | 27 (93.1) | 2 ( 6.9) | 0.436 |
| Illicit drugs use | Never | 240 (98.8) | 3 ( 1.2) |  |
|  | Less than monthly | 49 (90.7) | 5 ( 9.3) |  |
|  | Monthly or more | 12 (92.3) | 1 ( 7.7) | **0.004** |
| When did you last see a dentist? | Less than one year ago | 189 (97.4) | 5 (2.6) |  |
|  | Between 1 and 3 years ago | 85 (96.6) | 3 (3.4) |  |
|  | Between 3 and 5 years ago | 13 (92.9) | 1 (7.1) |  |
|  | Between 5 and 10 years ago | 8 (100) | 0 |  |
|  | More than 10 years ago | 6 (100) | 0 |  |
|  | Never | 1 (100) | 0 | 0.913 |

* Data from the Oral Health Study^4^

** We compared characteristics, lifestyle factors and sexual history stratified by CT/NG status using SAS version 9.4 (SAS, Cary, NC).

*** Current smokers were actively smoking, ex-smokers had stopped smoking at least one month earlier, and non-smokers had smoked 100 or fewer cigarettes in their life-time.

**** 1 Australian standard drink contains 10 grams of alcohol
